# Evaluation of the Effectiveness of the Adverse Drug Reaction Alert Card System in Preventing the Recurrence of Adverse Drug Reactions

**DOI:** 10.7759/cureus.64653

**Published:** 2024-07-16

**Authors:** Sarita Mulkalwar, Shantanu Chitale, Prachi Dandage, Shraddha Bapat, Abhijeet V Tilak, Sayali Patil

**Affiliations:** 1 Pharmacology, Dr. D. Y. Patil Medical College, Hospital and Research Centre, Pune, IND; 2 Pharmacology, D. Y. Patil University, School of Medicine, Navi Mumbai, IND

**Keywords:** adverse drug reactions (adr), healthcare professionals (hcp), adverse drug reaction reporting, adr monitoring, adr recurrence prevention, adverse drug reaction alert card

## Abstract

Introduction

Adverse drug reactions (ADRs) are among the leading causes of morbidity and mortality. It causes a significant prolongation of hospital stays, leading to an increased economic and infrastructural burden on the healthcare system. Thus, primary prevention will help in preventing recurrent ADRs. People are often unable to state whether they have suffered an ADR from a medicine or not. The patients also find it difficult to recall the offending drug. They seldom seem to carry any information that would warn others of their reactions. Thus, there was a need to introduce the ADR Alert Card. A pilot study was first conducted in 2018 to assess the feasibility of this card. All patients suffering from an ADR were thus provided an alert card. Following its implementation, there was a general acceptability regarding the potential of this card in ADR recurrence prevention among healthcare professionals (HCPs). Therefore, there is a need to assess the effectiveness of this card for ADR recurrence prevention.

Objective

This study aims to estimate the percentage of people who have shown the ADR Alert Card to their HCPs and benefited from it.

Methods

This was a prospective observational study, which was conducted at Dr. D. Y. Patil Medical College, Pune, from November 2022 to May 2024 and received approval from the Institutional Ethics Committee (IEC) before its initiation. All the patients who faced an ADR were given an ADR Alert Card by their HCP. All the patients to whom their HCP had given the card were part of this study. Any patient who suffered an ADR due to overdosage of medication was excluded from the study. After screening for inclusion and exclusion criteria, the data were analyzed using MS Excel (Microsoft Corporation, Redmond, Washington). A questionnaire was validated by professors in pharmacology, medicine, and community medicine. The patients were contacted through telephone conversations and provided with this questionnaire. They were asked questions regarding the ease of carrying the card, the benefit it provided them, whether they had shown it to their HCP, whether it helped them in an emergency, and their willingness to link it digitally. Their responses were recorded in Google Forms, and pie charts were generated.

Results

All 110 patients (100%) agreed that the ADR Alert Card was beneficial. Most (99, 90%) patients had shown the card to their HCP at their subsequent visit. The card helped 107 (97%) patients to describe their medical history easily. All the patients (110, 100%) agreed that carrying the card was easy, and most patients (95, 86%) agreed to recommend using the card to others. Additionally, most patients (79, 72%) were willing to link their card to their National Health ID. However, a small proportion of patients (28, ~25%) were skeptical whether they would link the card to the National Health ID or not. The card had helped 28 (25%) patients in an emergency. Approximately 11 (10%) patients had reported an ADR to the regulatory authority.

Conclusion

The patients welcomed this new concept to be inculcated in their daily lives as an effective means to enhance their healthcare. This study evaluates the number of patients who actually benefitted from using this card. It encourages patients to participate actively in their own healthcare. In an emergency situation, it proves to be a source of important health information. This study could lay the foundation for further research to prevent recurrent ADRs.

## Introduction

The World Health Organization (WHO) defines an adverse drug reaction (ADR) as “one which is noxious and unintended, and which occurs in doses normally used in humans for prophylaxis, diagnosis or therapy of disease, or for the modification of physiological functions” [1]. ADRs are among the leading causes of morbidity and mortality [2,3]. The rate of ADR reporting in India is less than 1%, while it is 5% globally due to a lack of awareness among patients and healthcare professionals (HCPs) regarding pharmacovigilance (PV) and ADR monitoring [4]. In the Indian setup, approximately 5%-25% of the admissions in the hospital are due to ADRs, and 6%-15% of hospitalized patients experience serious ADRs, causing a significant prolongation of hospital stay [4]. This leads to an increase in healthcare burden and thus adds to the infrastructural and economic stress of the already scarce resources in the healthcare setup [4].

Currently, the systems in place to report ADR in India are through the ADR reporting forms, reporting through a mobile-based ADR Pharmacovigilance Programme of India (PvPI) application, providing a toll-free helpline to report the ADR, ADR form drop boxes installed in hospitals, and voluntary reporting through mail or post. The pharmacovigilance systems in various countries, such as the USA, UK, Australia, and New Zealand, also include online reporting, submission of PDF or printed forms, mobile applications, and a toll-free helpline [5].

Recurrent ADRs are a recognized problem that leads to preventable patient distress. Taking into consideration the costs of ADR management, primary prevention will do a great deal to better utilize the limited resource provision in most healthcare systems [6-8]. People often cannot state whether they have suffered an adverse reaction to a medicine or are allergic to some medicine. Furthermore, they seldom seem to carry any information that would warn others of their reactions. In certain circumstances, this could have serious consequences. For example, Puranik reported a case of anaphylactic reaction caused by ceftriaxone, in which the patient experienced an ADR soon after its administration. If the patient had been aware of the ADR history beforehand, the situation may have been averted, and he would not have been given the medicine [9]. This vastly increases the chances of ADR recurrence in cases where it could have been easily prevented [8-11]. A study conducted by Jin and Min analyzed 85 case reports on ADR, which showed that 46 (54.12%) cases had unknown or idiosyncratic reactions, while 18 (21.18%) cases had a past ADR history [12]. According to the Consumer Protection Act (CPA), it can be stated that the legal liability of a recurrent ADR lies with the medical professional if he/she is aware of it. At the same time, the CPA also safeguards doctors if death is caused by any adverse reaction that is unknown or unpredictable [13,14].

To minimize or avoid the recurrence of ADR, we have introduced our version of the ADR Alert Card. We have implemented the ADR Alert Card system in our tertiary healthcare center. We have a copyright certificate from the Copyright Office, Government of India (Registration No. L-111544/2022, dated January 31, 2022). Providing an ADR Alert Card to the patient will make them more aware of their ADR status. This may also make them more proactive and responsible toward providing a profound history to the HCP and thus be a part of their own healthcare management. The patients will be able to state their drug sensitivity and carry confirmatory evidence. This system will probably cause minimal disruption to the ongoing practice and will strengthen the patient safety profile while prescribing. Several states in India and several countries worldwide have developed their own versions of the ADR Alert Card to date [15-21].

A pilot study was first conducted in 2018 at Dr. D. Y. Patil Medical College, Hospital & Research Centre, Pune, to assess the feasibility of the ADR Alert Card concept. This was well-received by both patients and clinicians since it educated patients on their drug reactions while serving as a physical record. Feedback from both clinicians and patients motivated the management to implement the ADR Alert Card system in the institute. Furthermore, an online, descriptive, cross-sectional survey was conducted for a duration of six months from March to August 2021 across India to collect the opinion of HCPs and evaluate the current status of ADR recurrence prevention measures. However, the survey is still awaiting publication (IN PRESS). The study asked HCPs about the practices they followed for ADR recurrence prevention and suggestions. Most HCPs (76.7%) believed that ADRs could be prevented if patients provided proper ADR history. This is practically not feasible as it is difficult for the patients to remember the name of the offending drug that caused the ADR. Some HCPs (21.7%) mentioned the ADRs in the patient's file, but this is not feasible as the patients do not carry their files constantly with them. Some HCPs created health tags, while some stressed the need to educate patients and their relatives about the ADR. However, if the patient carries an ADR Alert Card every time they visit an HCP, the problem of ADR recurrence could be curbed to a greater extent. The patient will just have to produce the ADR Alert Card while providing their history to the HCPs. This study depicted the willingness of HCPs (98%) to inculcate the ADR Alert Card system in their own institute. Following the implementation of the ADR Alert Card, there was a general acceptability regarding the potential and effectiveness of this card in ADR recurrence prevention.

Thus, there was a need to evaluate the effectiveness of this ADR Alert Card for ADR recurrence prevention as a parallel route to strengthening the pharmacovigilance (PV) system and, hence, our country's healthcare system.

## Materials and methods

This was a prospective observational study that was conducted from November 2022 to May 2024 at Dr. D. Y. Patil Medical College, Hospital and Research Centre, Pimpri, Pune, after obtaining approval from the Institutional Ethics Committee (IEC) (IESC/PGS/2022/201).

Inclusion criteria

The hospitals and HCPs who were willing to implement the ADR Alert Card system in their own clinic/hospital/institute were provided with ADR Alert Cards©(Registration No. L-111544/2022, dated January 31, 2022) (Appendix 1). Informed consent for telephonic calls was obtained at the end of the first and sixth months of follow-up. Patients who have been given an ADR Alert Card by these hospitals/HCPs were included in this study. The patients suffering from preventable ADRs (allergic reaction, idiosyncratic reaction, etc.) were provided with an ADR Alert Card.

Exclusion criteria

Any patient suffering from an ADR due to overdosage of medication was excluded from the study.

Study protocol

The ADR Alert Card was designed considering its resourcefulness as a source of pre-validated medical information about the patient. The ADR Alert Card was intentionally designed to have sizes similar to those of a credit card, allowing for easy and convenient integration into everyday life by fitting seamlessly into a wallet or purse.

The hospitals and HCPs were asked to sign the copyright form (Appendix 2) before using the ADR Alert Card. HCPs counseled the patients about the continued use of this card. The front side of the ADR Alert Card has multiple details, such as the individual's name, contact information, emergency contact number, blood type, and the drug reaction reporting helpline provided by PvPI. The back side contained details regarding the name of the medication, the adverse reaction caused by the medication, and the date of onset of the reaction (Appendix 1). They were asked to send a photo of the ADR Alert Card to the number provided to them. The HCPs were provided a letter (Appendix 3) to guide them regarding the effective use and implementation of the ADR Alert Card.

The patients suffering from preventable ADRs (allergic reaction, idiosyncratic reaction, etc.) were provided with an ADR Alert Card. HCPs instructed the patients to keep the card with them 24/7 to prevent further recurrence. They were also advised to keep the photo of the card on their mobile phones. Thus, patients were possibly protected from re-exposure to the offending drug whenever possible.

The information on these ADR Alert Cards received from HCPs was tabulated (Appendix 4). Thus, follow-up (in the form of a Google questionnaire and telephone interviews) (Appendix 5) was conducted twice, i.e., in the first and sixth months of the study.

Measured outcomes

The study aims to estimate the percentage of people who have shown the ADR Alert Cards to their HCP, to estimate the percentage of people carrying their ADR Alert Card during the first (one month) and second (sixth month) follow-up, to estimate the percentage of people who get benefited by the use of ADR Alert Card, and to estimate the percentage of people willing to link their ADR Alert Card to their Ayushman Bharat Health Account (ABHA).

Follow-up

The study comprised two follow-up periods. The first follow-up, conducted after one month, checks whether the patients still carry their ADR Alert Card. The second follow-up, performed in the sixth month, involves administering the questionnaire mentioned below. Additionally, the card's availability with them was checked again.

Statistical analysis

The data collected from telephone interviews were entered into Google Forms, and pie charts were generated. Using MS Excel (Microsoft Corporation, Redmond, Washington), the data of the ADR Alert Card was preserved (Appendix 4). Tables were created using the numbers of ADRs within the affected drug class (N) and percentage expressions.

## Results

All 110 patients met the inclusion criteria, and their data were analyzed. These data were evaluated to check the effectiveness of the ADR Alert Card and the benefits it may have provided. The study population comprised 70 (63.6%) female and 40 (36.4%) male patients. Adults (18-65 years) comprised the most affected age group, accounting for 89 (81%) patients. The observations of this study are presented in Table 1.

**Table 1:** Results based on the questionnaire ADR: adverse drug reaction

Questions	Yes (N (%))	No (N (%))	Maybe (N (%))
Do you think carrying an ADR alert card is beneficial?	110 (100)	-	-
Have you shown the ADR card to your healthcare professional till now?	99 (90)	11 (10)	-
Has the ADR alert card helped you to describe your medical history easily?	107 (97)	3 (2.7)	-
Is it easy for you to carry the ADR alert card easily?	110 (100)	-	-
Will you recommend its usage to other people?	95 (86.4)	-	15 (13.6)
Are you willing to link it to the Ayushman Bharat Health Account in the future?	79 (71.8)	3 (2.7)	28 (25.5)
Has it helped you in an emergency situation?	28 (25.5)	82 (74.5)	-
Have you reported any ADR to the regulatory authority?	11 (10)	99 (90)	-

All patients (N=110) agreed that the ADR Alert Card is beneficial. The majority of them had shown the ADR Alert Card to their HCP. The ADR Alert Card helped most patients describe their medical history easily. All the patients concurred that carrying the card was easy for them. Most of the patients agreed to recommend the use of the ADR Alert Card to other people. Most patients were willing to link their ADR Alert Card to the National Health ID, i.e., Ayushman Bharat Health Account. A small proportion of patients were skeptical whether they would link the card to the National Health ID. The ADR Alert Card had helped some of the patients in an emergency situation, while most patients had not yet faced an emergency situation and thus had not used the ADR Alert Card in an emergency. Many patients had not yet reported an ADR to the regulatory authority, while others who reported an ADR were mainly HCPs. All the patients carried the ADR Alert Card with them during the first and sixth months of follow-up.

Based on information collected through all the received ADR Alert Cards from HCPs, the following information regarding the number of ADRs and the percentages of causative drugs and their related information was tabulated in Table 2.

**Table 2:** Patients suffering an ADR with respect to the affected class of drugs, N (%) NSAID: non-steroidal anti-inflammatory drugs; DMARDs: disease-modifying antirheumatic drugs; CNS: central nervous system; BZDs: benzodiazepines; I.V.: intravenous; TCA: tricyclic antidepressants; OHA: oral hypoglycemic agent; JAK: Janus kinase; ADR: adverse drug reaction

Drug Class	N (%)
Antimicrobial drugs	59 (53.6)
Beta-lactam antibiotics	32 (29)
Cephalosporins	25 (22.7)
Penicillin	4 (3.6)
Amoxicillin	3 (2.7)
Aminopenicillin	3 (2.7)
Quinolones	7 (6.3)
Ciprofloxacin	3 (2.7)
Norfloxacin	1 (0.9)
Ofloxacin	3 (2.7)
Tetracyclines-Doxycycline	2 (1.8)
Macrolides-Azithromycin	2 (1.8)
Aminoglycosides	6 (5.4)
Vancomycin	4 (3.6)
Amikacin	2 (1.8)
Glycopeptide-Teicoplanin	2 (1.8)
Antifungal-Itraconazole	2 (1.8)
Anti-protozoal-Azole	3 (2.7)
Miscellaneous	15 (13.6)
Multivitamins	3 (2.7)
Radiocontrast agents	2 (1.8)
Expectorants	2 (1.8)
Mucolytics	1 (0.9)
I.V. fluids	2 (1.8)
Alpha-keto analogue	1 (0.9)
Anti-diarrheal	1 (0.9)
Steroids	1 (0.9)
TCA	1 (0.9)
Spasmolytic	1 (0.9)
NSAIDs	12 (10.9)
Paracetamol	5 (4.5)
Ibuprofen	4 (3.6)
Diclofenac	2 (1.8)
Aspirin	1 (0.9)
DMARDs-Sulfasalazine	5 (4.5)
Hematinic	5 (4.5)
Anti-emetics	4 (3.6)
Ondansetron	3 (2.7)
Metoclopramide	1 (0.9)
Anti-cancer drugs	4 (3.6)
Opioid analgesics	2 (1.8)
Tramadol	1 (0.9)
Fentanyl	1 (0.9)
CNS depressants	2 (1.8)
Barbiturates	1 (0.9)
BZD	1 (0.9)
Anti-hypertensive	1 (0.9)
Anesthetics-Ketamine	1 (0.9)

The most common group of drugs with which ADRs were reported were antimicrobials, miscellaneous drugs, and non-steroidal anti-inflammatory drugs (NSAIDs). Among antimicrobials, the patients faced the majority of ADRs with cephalosporins. ADRs with miscellaneous drugs comprised of multivitamins, mucolytics, expectorants, radiocontrast agents, and IV fluids. ADRs with NSAIDs were commonly seen with paracetamol and ibuprofen.

## Discussion

An efficient ADR reporting system is the backbone of reliable practice and a measure of progress toward achieving drug safety. Improvements in the efforts and system changes of ADR reporting systems should be targeted toward reductions in the likelihood of drug reactions in future patients [15]. Preventable adverse drug reactions (PADRs) are ADRs caused by pharmaceutical errors, including acts of omission or commission, improper medicine/dose/timing, prescribing a medication to a patient with a known allergy, inadequate monitoring, or other errors [22,23].

The ADR Alert Card is a simple tool that enables patients to carry their important medical history. The ADR Alert Card has the following advantages as compared to the available options [5]: (1) It holds the patient responsible for the prevention of the recurrence of ADR by making them aware of their own ADR status and giving them the responsibility of showing their ADR Alert Card to the HCP, thus reducing their burden; (2) in an emergency, this card can be used as a source of important health information. The patient’s blood group, history of major illness, and adverse reaction could be beneficial for the health professional to decide on a further course of action (Appendix 1); (3) the emergency contact number given on the card can help in notifying the relatives (Appendix 1); (4) it is easy to carry in the wallet/purse/pocket. It gives a sense of empowerment to the patient to take an active part in his own healthcare. Most HCPs generally write ADRs on the patient's file, but the patient does not carry the same file whenever he goes to any hospital. Also, the patients do not carry the file with them 24/7; (5) the card can be made in different vernacular languages to make it user-friendly; and (6) there will be an improvement in ADR reporting as the patient himself can report it on the PvPI number given on the card (Appendix 1).

The ADR Alert Card may be an efficient means to enhance awareness among HCPs about ADR reporting. This may strengthen the ADR reporting culture of the institution and improve its contribution to the PvPI/National Pharmacovigilance Programme. Patient involvement is equally important and contributory, which could strengthen the ADR reporting being captured within the system. Moreover, in our country’s low-budget scenario, where there is a lack of electronic health records (EHRs) at the institutional as well as national database level as well as a hurdle of patient literacy, this card will be a turning point to combat the problem of ADR recurrence [24].

The trend of ADRs concerning the demographic parameters as well as drug categories showed that the rate of ADR in female patients (70, 63.6%) was found to be higher than that in male patients (40, 36.3%), which corresponds to the findings of Thakare et al. and Sharma et al. [25,26]. In a retrospective study conducted by Thakare et al., ADRs were analyzed according to year, gender, age group, pharmacological class, and department [25]. They concluded that sensitization programs can help improve the rate and quality of ADR reporting. The risk of developing an ADR is 1.5-1.7 times greater in female patients than in male patients [27]. Although the precise reason for this increased risk is unknown, it may be connected to variations in hormonal, immunological, and pharmacokinetic characteristics associated with gender [27]. Adult patients or the breadwinner group was the age group most commonly impacted in our study. This finding is consistent with a recently released study by Thakare et al. and Sharma et al. [25,26].

The pharmacological drug classes implicated in causing ADRs show that antimicrobials (59, 53.6%) caused the maximum number of ADRs (Table 2), which was consistent with the findings of Thakare et al. and Sharma et al. [25,26]. Most patients (25, 22.7%) were affected by cephalosporins, the most commonly affected drug class among the antimicrobials (Table 2). This was consistent with the findings reported by Daulat et al. [28]. This prospective observational study collected ADR reports from 130 patients to assess their causality, severity, and preventability per the standard scales. Antimicrobials (88, 68%) were the drug group most commonly involved in ADRs [28]. Patients should not be deprived of the most effective antimicrobial solely based on the history mentioned on the ADR Alert Card. For example, a patient allergic to ampicillin can be considered for another penicillin or cephalosporins, depending on the severity of their allergy.

Similarly, third- or fourth-generation cephalosporins may be administered to a patient with a history of allergy to first- or second-generation cephalosporins, provided that the allergy is not severe. Each case should be evaluated individually, considering the patient's allergy history, the severity of previous reactions, and the availability of alternative antibiotics. The ADR Alert Card will streamline the history-taking process for HCPs, allowing them to gather necessary information more quickly. Cephalosporins have a higher risk of cross-reactivity, with estimates ranging from 1% to 8% [29,30]. Studies suggest that third- and fourth-generation cephalosporins can be given as they have lower chances of cross-sensitivity [28,29]. In short, if a patient is allergic to one cephalosporin, it does not necessarily mean they cannot receive another cephalosporin [29,30]. The likelihood of cross-reactivity depends on the specific cephalosporin generation and side chain. Clinicians should carefully evaluate each patient’s history and allergy profile to determine the best course of treatment [29,30]. Drug history should be asked in detail, and the HCP should be alert while reusing the drug responsible for allergy and should go for skin testing, graded challenge, and consideration of alternative antibiotics. The ADR Alert Card helps in raising an alarm regarding the use of the same drug or a drug from the same group.

The responses and feedback from patients through telephone interviews were collected to check the feasibility of carrying the card and the benefits they experienced as a result. In the first month of follow-up, all the patients (110, 100%) had kept the ADR Alert Card with them. This signifies that the patients were convinced that it was important to carry the card. Some patients also described how they kept their ADR Alert Card safely, i.e., in their wallet/purse. The parents of some patients attached their child’s ADR Alert Card to their school diary to provide important information to the clinician in the absence of the parents.

At the end of the sixth month, these patients were contacted again to check the availability of the ADR Alert Card. A questionnaire was administered to assess the usefulness of this card. At first, they were asked whether they found the ADR Alert Card beneficial. To this, there was a 100% agreeability. This shows that patients are ready to accept and adapt to a new change, possibly contributing to improving their healthcare. Most patients (99, 90%) had shown the ADR Alert Card to their HCP during their subsequent visits/follow-ups. This depicts a general sense of awareness among patients to actively participate in their own healthcare and provide proper history. Most patients (107, 97%) agreed that the ADR Alert Card has helped them describe their medical history easily. This depicts that the ADR Alert Card can act as an effective means to assist patients in providing their medical history. In turn, it can also ease the HCP's decision on the right drug for the patient. There was a general agreeability among all the 110 patients (100%) that they could easily carry the card and keep it with them. This shows that any new health-related intervention that causes the least disruption in patients’ lifestyles is usually well-received and acknowledged. Furthermore, 95 (86%) patients also agreed that they would recommend its use to others. This depicts a general sense of willingness to accept and adapt to a new change in healthcare and also spread its awareness among everyone (Table 1).

In a country such as India, the use of digital technology has become crucial for daily operations and obtaining life-sustaining benefits in day-to-day life [29]. The patients were asked about their willingness to link their physical ADR Alert Card to the National Health ID. In this era of digitization, 79 (~72%) patients were willing to link their ADR Alert Card to the National Health ID. This shows the patients' futuristic outlook in digitizing their health records so they do not have to keep any physical card handy while visiting HCPs. Approximately 28 (25%) patients were skeptical about linking their card and getting it digitized (Table 1). They expressed concerns over providing their private health details on the internet and, in turn, voiced their worries over data breaches and confidentiality. The most recent event in India regarding the violation of healthcare data was the AIIMS ransomware attack on November 23, 2022, where the health data of millions of people were exposed [30].

Furthermore, after giving the ADR Alert Card, we asked the patients whether it was useful to them in an emergency to date. We found that this card has helped 28 (~25%) patients in an emergency where they had shown this ADR Alert Card. Some instances were when this card was shown to the casualty medical officer (CMO) while visiting the casualty. In this way, the offending drug was outrightly avoided by the CMO as the patient or their relatives were aware of the patient’s drug history. Some found it helpful in times of urgent blood transfusion as this card provides information regarding the patient's blood group. Most patients (82, ~75%) had not yet faced an emergency to use this card (Table 1).

The patients were asked whether they had reported any ADR to the regulatory authorities. Most of the patients (99, 90%) had not reported any ADR to the regulatory authority, nor were they aware of it. This information about ADR reporting through the PvPI helpline should be communicated to the patients by the HCPs while giving the ADR Alert Card. These findings are consistent with the study conducted by Fossouo et al., in which they found that only 11 (10.4%) patients were aware of ADR reporting. This study was conducted in Australia to review how consumers and HCPs participate in ADR monitoring and reporting. They found that giving feedback to patients on their ADR reports and including them in the ADR management process by giving them ADR Alert Cards will increase patient happiness and raise patient awareness [31]. The remaining patients (11, 10%) were mainly comprised of HCPs who had suffered an ADR and were aware of ADR reporting (Table 1).

A question arises whether we need to give these ADR Alert Cards to everyone who faces a reaction. The distribution of ADR Alert Cards should also be done mindfully. It should be given to patients with an allergic or idiosyncratic reaction. For example, some patients are very sensitive to diclofenac even on normal doses and suffer from kidney injury, while some patients are sensitive to antipsychotics and have extrapyramidal side effects. If a patient develops a rash even on low-dose ampicillin/penicillin, it should be considered an ADR. Such patients should be given an ADR Alert Card. On the other hand, if a patient on NSAIDs who had not taken prior H2 blocker/PPI suffers from gastritis, it should be considered as a side effect and not an ADR. The way to differentiate this is to give exclusive attention on a case-to-case basis and then give an ADR Alert Card.

A limitation of the study is that the ADR Alert Card, given to the patients, is a physical card that may be prone to be misplaced or lost, potentially compromising its benefit to the patient. Thus, the need to link it digitally further increases. Another limitation is that if the HCPs are not appropriately counseled regarding the distribution of the ADR Alert Card to the right patient, as discussed above, there is a possibility of overinclusion and over-usage of this card, which might not be helpful. To optimize the utilization of this ADR Alert Card, it is imperative for the healthcare community to diligently educate patients about recurring ADRs by utilizing this card. Both patients and HCPs should undergo awareness and training initiatives to enhance their knowledge about this card, leading to increased utilization.

Implementing ADR Alert Cards in the future could result in decreased healthcare expenses for both our country's healthcare system and individual patients by preventing the recurrence of ADRs. There could be a standard practice across all hospitals where HCPs compulsorily ask the patients about their ADR history. The ADR Alert Card could be made compulsory across all the hospitals in India. The ADR Alert Card might be digitized and integrated with the National Health ID, allowing for the generation of a QR code.

## Conclusions

Overall, the patients were receptive to the introduction of this new concept into their daily lives as an effective means to enhance their own healthcare. This card will act as an adjuvant to strengthen ADR reporting. It will encourage the patients to take active participation in their own healthcare. The study examined the use and effectiveness of this card for patients to prevent recurrent ADRs. Thus, it was useful to check the number of patients who benefited from using the ADR Alert Card. In an emergency situation, it proved to be a source of important health information. This study could lay the foundation for further research to prevent recurrent ADRs.

## Figures and Tables

**Figure 1:**
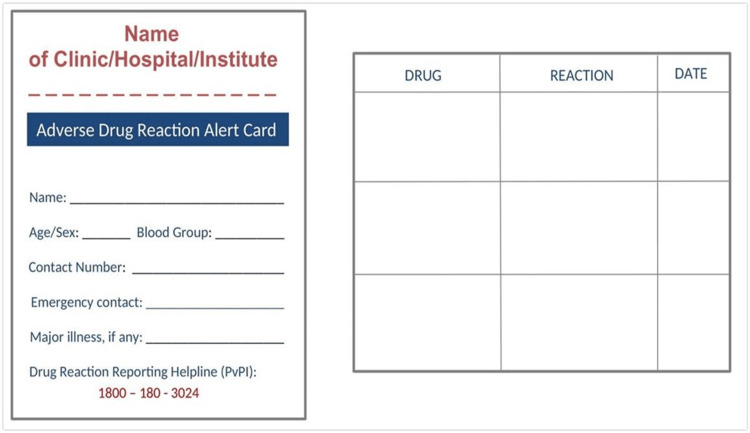
The front and back views of the ADR Alert Card ADR: adverse drug reaction; PvPI: Pharmacovigilance Programme of India

**Figure 2:**
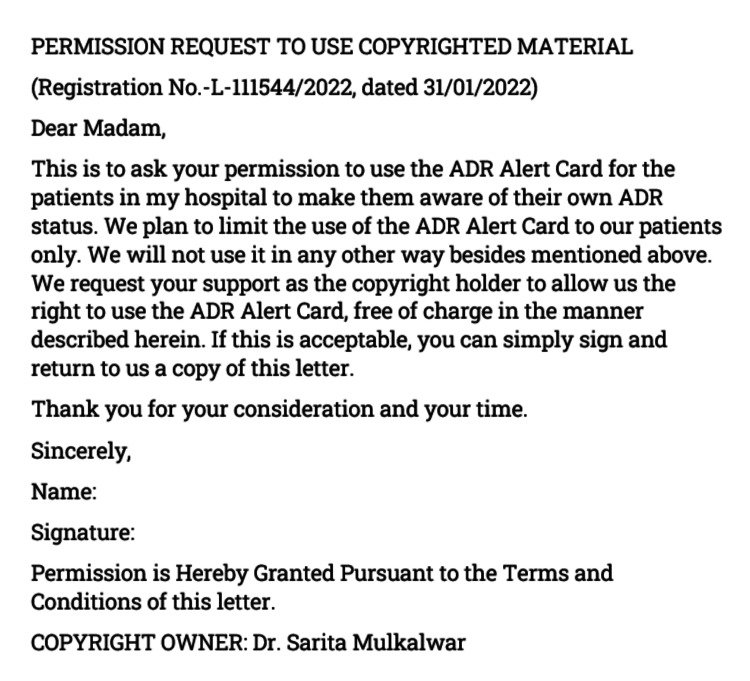
Permission request to use the ADR Alert Card (Copyright Form) ADR: adverse drug reaction

**Figure 3:**
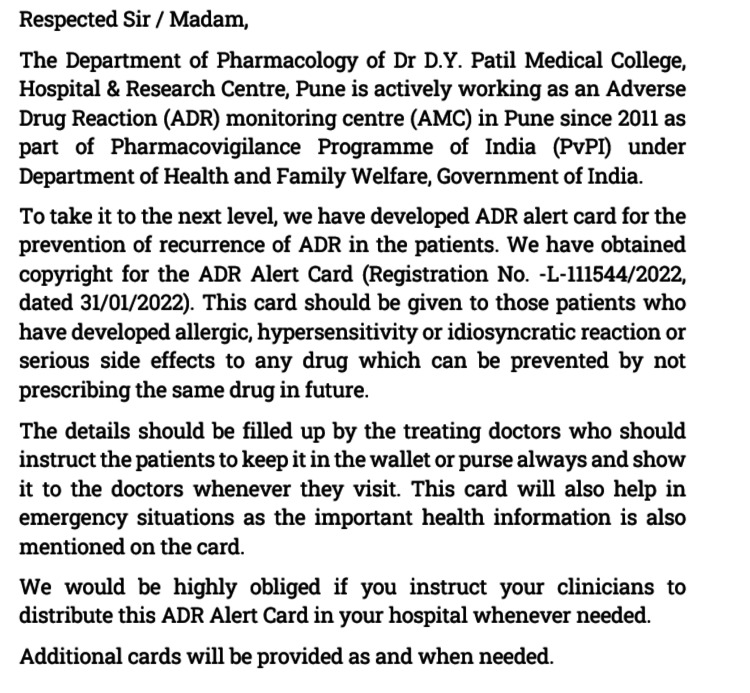
Instructions to HCPs regarding the use of the ADR Alert Card ADR: adverse drug reaction; HCPs: healthcare professionals; AMC: ADR Monitoring Centre

## Appendices

Appendix 1

Appendix 2

Appendix 3

Appendix 4

**Table 3:** Data on the ADR Alert Cards as received from the HCPs ADR: adverse drug reaction; HCP: healthcare professional

Sr No.	Name	Age	Sex	Blood Group	Contact No./ Emergency Contact No.	Major Illness (If Any)	Drug Taken	Reaction	Date	Follow-Up (1st / 2nd)	ADR Card still carrying / lost? (1st month/ 6th month)

Appendix 5

**Table 4:** Questionnaire for follow-up ADR: adverse drug reaction

Questionnaire
1) Do you think carrying an ADR Alert Card is beneficial?
2) Have you shown the ADR card to your healthcare professional till now?
3) Has the ADR Alert Card helped you to describe your medical history easily?
4) Is it easy for you to carry the card?
5) Will you recommend its usage to other people?
6) Are you willing to link it to your Ayushman Bharat Health Account in the future?
7) Has it helped you in an emergency situation?
8) Have you reported any ADR to the regulatory authority?
